# Scapular kinematic reconstruction – segmental optimization, multibody optimization with open-loop or closed-loop chains: which one should be preferred?

**DOI:** 10.1080/23335432.2017.1405741

**Published:** 2017-12-15

**Authors:** Benjamin Michaud, Sonia Duprey, Mickaël Begon

**Affiliations:** a Laboratoire de simulation et de modélisation du mouvement, Département de kinésiologie, Université de Montréal, Laval, Canada; b Univ Lyon, Université Claude Bernard Lyon 1, IFSTTAR, LBMC UMR_T9406, F69622, Lyon, France; c Research Center, Sainte-Justine Hospital, Montreal, Canada

**Keywords:** Keywords, Scapula, kinematics, segmental optimization, multibody kinematics optimization (MKO)

## Abstract

Several numerical approaches have been developed to address the soft tissue artefact, such as the segmental optimization and multibody kinematics optimizations using either open-loop or closed-loop chains. However, it is still not clear which method is the most efficient for scapular kinematics reconstruction. In this study, six kinematic models were compared to a silver standard, i.e. a scapula palpator, during a series of 55 postures with maximal amplitudes of both the arm and scapula performed by 15 subjects. The most accurate approaches were the multibody optimization with a closed-loop chain and the segmental optimization. They provided averaged scapula misorientations of 14.9 ± 6.7° and 16 ± 7.1, respectively. Regarding the closed-loop chain integrating a point-to-ellipsoid scapulothoracic joint, the ellipsoid providing the most accurate results was a thorax-size ellipsoid fitting the area browsed by the scapula. Eventually, considering the high implementation costs of a multibody kinematics optimization, the segmental optimization could be considered as appropriate for scapular kinematics reconstruction.

## Introduction

The scapulohumeral rhythm – i.e. the kinematic interaction between the scapula and the humerus – can be associated with diverse shoulder conditions (Kibler 2003) and therefore can be used as an indicator of shoulder disabilities. To calculate the scapulohumeral rhythm, both the scapular and the humeral kinematics must be acquired. However, the scapular kinematics is not trivial to measure. Indeed, non-invasive methods based on skin markers may be ineffective due to soft tissue artefacts (Cappozzo et al. 1996). The large amount of muscles covering the scapula and the sliding movements of this bone under the skin can generate artefact up to 90 mm (Matsui et al. 2006). Furthermore, most of the studies focusing on scapula kinematics investigate classic humeral elevation motions (i.e. flexion or abduction) (Lempereur et al. 2014), while scapula range-of-motion may be larger during sport or daily-living activities. Thus, to assess the reliability of a measurement method dedicated to scapula kinematics, tasks requiring large scapula amplitude could bring a more thorough insight.

To address the scapula soft tissue artefact issue, experimental and numerical solutions have been proposed. As for experimental approaches, the use of a scapula palpator (Pronk and van der Helm 1991; Johnson et al. 1993) can give a precise and valid measure of the scapular kinematics with a reconstruction error as low as 2° (de Groot 1997; Lewis et al. 2002; Lempereur et al. 2010). The scapula palpator, also called scapula locator, is a triangular device that is manually adjusted on the scapula geometry such that its three extremities touch three scapula anatomical landmarks, namely angulus inferior, trigonum spinae and angulus acromialis. Since it has to be adjusted before each measure, the acquisition frequency is limited to approximately 1 Hz for trained experimenters (Meskers et al. 1998), compromising its use in dynamic applications (Meskers et al. 2007). In parallel to the development of the scapula palpator, methods based on skin markers have been improved to acquire dynamic movements: the use of an acromial marker cluster (van Andel et al. 2009; Duprey et al. 2015) combined with a double-calibrate procedure is currently one of the most effective method (Cereatti et al. 2015). However, it is still limited to low arm elevations, under 100° (Lempereur et al. 2014) and movements in a unique plane.

As for numerical solutions, optimization approaches were developed to reduce the soft tissue artefact at the scapula: the segmental and the multibody kinematics optimizations. The segmental optimization consists in minimizing the deformation of a cluster of markers located on a given segment. This approach leads to errors up to 15° while reconstructing the scapula kinematics during arm elevations (Prinold et al. 2011; Lempereur et al. 2014). The error for movements involving largely the scapula range-of-motions (i.e. daily-living or sport movements) remains unknown. The multibody optimization relies on the use of a kinematic chain: the kinematic is reconstructed by minimizing the distances between the measured marker positions and the marker positions predicted by the kinematic chain. This approach leads to error as low as 4° in arm flexion and abduction of maximal amplitude (Charbonnier et al. 2014). However, the kinematic models of the upper limb are numerous and various (Duprey et al. 2016) and do not systematically integrate the scapula or the scapulothoracic joint. Thus, it is not possible to generalize this low error value. The scapulothoracic joint is commonly modelled as a contact of one to three points of the scapula on an ellipsoid representing the thorax (van der Helm 1994; Garner and Pandy 1999; Maurel and Thalmann 1999). Indeed, *ex vivo* measurements showed that the scapula follows a rounded path around the thorax (Klein Breteler et al. 1999). However, when it comes to *in vivo* postures or motions, muscles contractions and contact forces may alter such rounded scapula paths. To account for this altered scapula path and also adapt to the various shapes of the thorax in the population, the ellipsoid must be personalized in terms of size and position. So far, two ellipsoid sizes were used, one where the thorax is modelled as a whole (Bolsterlee et al. 2014), resulting in a large ellipsoid, and one where the thorax is modelled as a half torso (Garner and Pandy 1999; Seth et al. 2010; Prinold and Bull 2014) resulting in a lung-sized ellipsoid. In these methods, the ellipsoid is scaled to fit positions of markers (essentially markers on the thorax) from one (Bolsterlee et al. 2014) or several (Prinold and Bull 2014) static poses. Another interesting approach could be to fit the ellipsoid based on scapula marker positions from several static poses so that the ellipsoid parameters would account for the whole area where the scapula can be located. However, to date, there is no consensus on the best way to generate the ellipsoid needed to model the gliding plane of the scapulothoracic joint.

Hence, currently, it remains unclear in the literature whether the scapula kinematics would benefit or not from the integration of a scapulothoracic joint. This raises the following questions. Could a segmental optimization approach be sufficient or is a multibody optimization necessary? In the case of a multibody optimization approach, should a closed-loop chain (integrating a scapulothoracic joint) be preferred over an open-loop chain? And finally, if a scapulothoracic was to be used, which ellipsoid definition should be favoured?

The objective of this paper is to determine which numerical method(s) among segmental and multibody (open- or closed-loop) optimization provide(s) the most accurate scapular kinematics in large ranges-of-motion of both the scapula and arm. Movements implying large ranges-of-motion of the scapula and high arm elevations will be investigated. A secondary objective was to test the effect of the arm elevation angle on the models’ accuracy.

## Materials and methods

### Experiments

**Figure 1.**
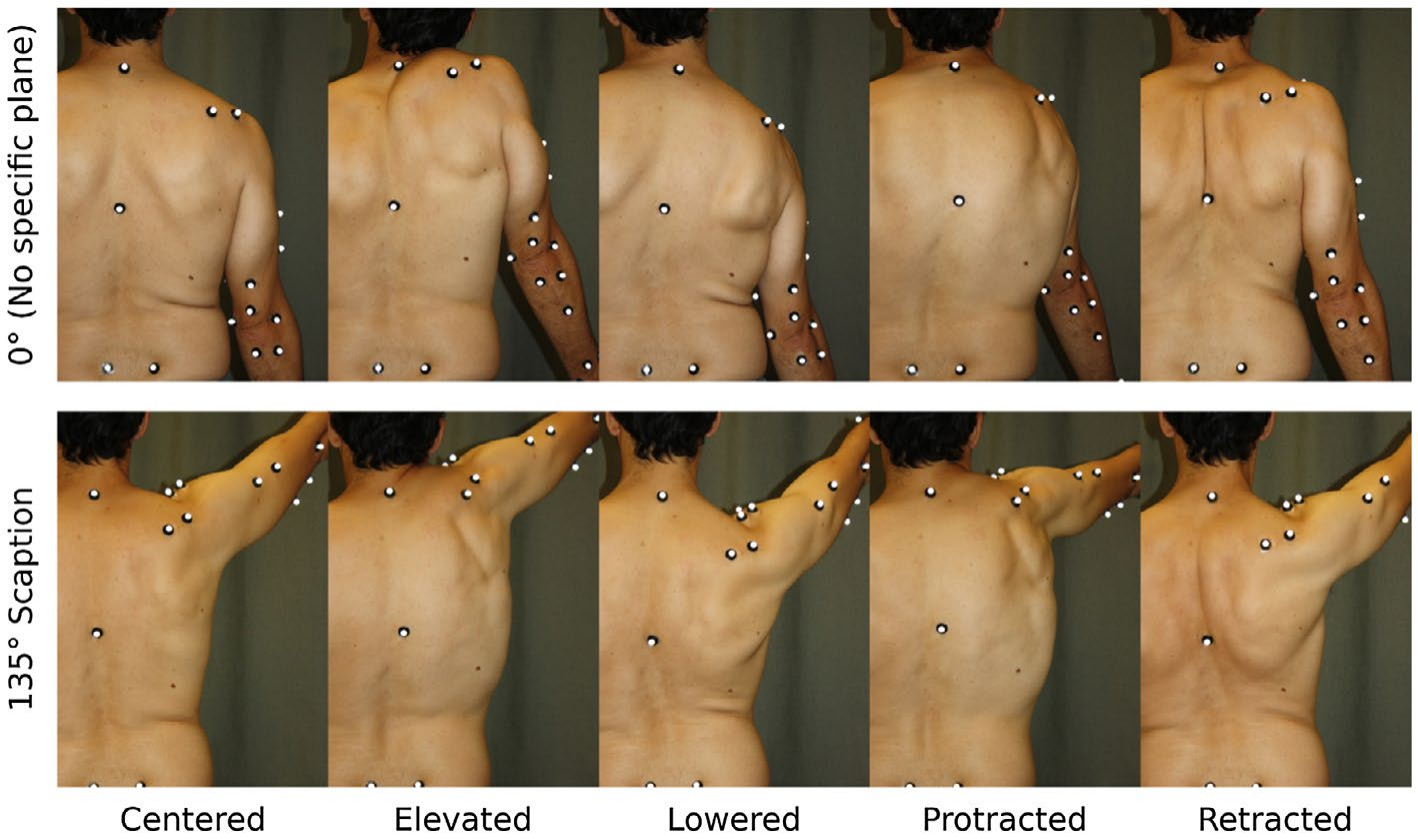
A participant equipped with reflective markers performing 10 of the 55 shoulder poses. Each column stands for a scapula pose (Centred, Elevated, Lowered, Protracted, Retracted) while the two rows correspond to two arm elevations (0° and 135° of flexion in the scapular plane).

Ten males (26 ± 5 years, 175 ± 9 cm and 71 ± 9 kg) and five females (24 ± 2 years, 161 ± 8 cm and 68 ± 8 kg) with no shoulder pain history took part in this study after giving their informed consent (ethics 14-110-CERES-D, *Université de Montréal*, Canada). The participants were equipped with 43 reflective markers (including 35 technical markers for the multibody optimization) put on their thorax and upper limb skin in line with the Jackson et al. (2012) marker set. Two additional geometrical markers were placed on the seventh cervical vertebra and on the most lateral point of the thorax to build geometrical ellipsoids (Bolsterlee et al. 2014; Prinold and Bull 2014). The reflective markers trajectories were collected at 100 Hz using an 18-camera Vicon™ optoelectronic motion analysis system (Oxford Metrics Ltd. Oxford, UK) during the following trials. Furthermore, a scapula palpator, adjusted on the geometry of each participant’s scapula, was used to measure the reference scapula kinematics (silver standard; Figure 1).

**Figure 2.**
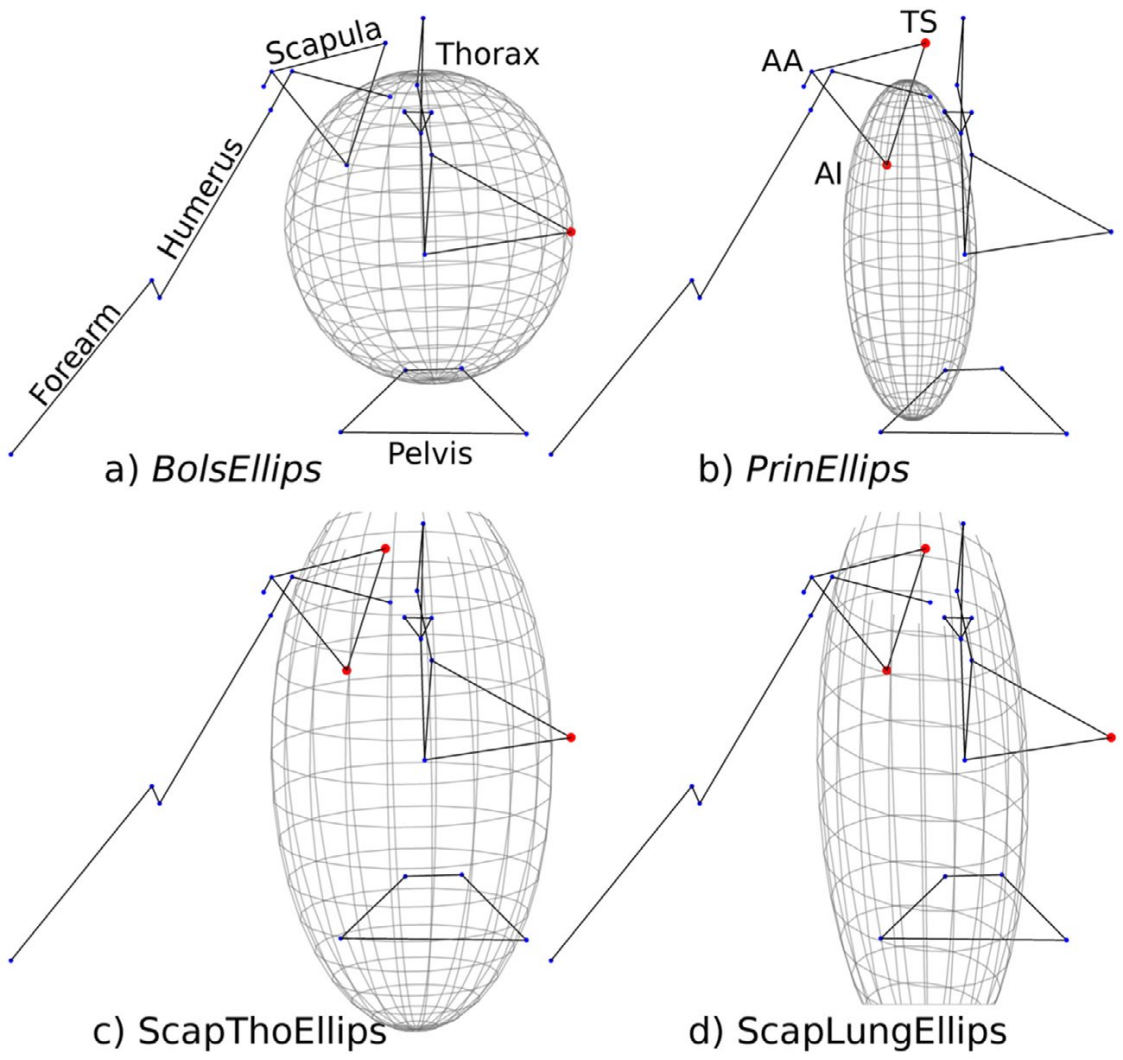
Illustration of the four kinematic chains integrating ellipsoids: (a) *BolsEllips*. (b) *PrinEllips*. (c) *ScapThoEllips*. (d) *ScapLungEllips*. The same scale was used for each ellipsoid. AA. AI and TS stand for Angulus Acromialis. Angulus Inferior and Trigonum Spinae.

First, the participants were asked to maintain several postures for a few seconds: an anatomical posture and three arm attitudes described in Prinold and Bull (2014) as (1) at rest with the arms by the side, (2) arms horizontal at 45° to the coronal plane and (3) arms at subject’s maximal elevation. Then, they were asked to perform dedicated movements in line with Begon et al. (2007) recommendations to functionally locate of the pelvothoracic and wrist joint centres as well as the elbow joint axis. Following these preliminary acquisitions, the participants were instructed to perform 55 poses resulting from the combination of 11 arm (thoracohumeral) attitudes with 5 poses of the scapula performed at maximal amplitude. The 11 arm attitudes were: (1) arm along the body (termed as 0°); (2) arm fully elevated (180°); (3–5) arm flexed at 45°, 90° and 135° in the sagittal plane; (6–8) arm flexed at 45°, 90° and 135° in the scapular plane; (9–11) arm abducted at 45°, 90° and 135°. The thoracohumeral angle was initially adjusted by the experimenter using a goniometer. The five scapula poses were: (1) centred; (2) elevated; (3) lowered; (4) protracted and (5) retracted (Figure 2). Each pose was maintained until the experimenter had palpated the scapula bony landmarks (angulus inferior, trigonum spinae and angulus acromialis) to accurately position the scapula locator. In this phase, the reflective markers on the three bony landmarks were removed. To avoid any muscle fatigue, participants could rest as long as they needed between the poses.

### Data processing

Kinematic data and ellipsoid parameters were expressed in the thorax reference frame (where *x* is along the medio-lateral direction, *y* along the infero-superior direction and *z* along the antero-posterior direction).

#### Kinematic chains

The functional movements were used in combination with SCoRE and SARA algorithms (Ehrig et al. 2006, 2007) to locate the pelvothoracic and wrist joint centres and the elbow joint axis. As recommended by Michaud et al. (2016), sternoclavicular, acromioclavicular and glenohumeral joint centres were located using anatomical or predictive methods. Locations of joint centres and anatomical makers served to define joint coordinate systems of the segements in the anatomical pose position (Jackson et al. 2012).

Six kinematic models were built (Table 1). The first included only the scapula, the second was an open-loop chain, while the four other kinematic chains included a scapulothoracic joint based on a point-to-ellipsoid contact. Due to the absence of consensus in the literature on the best ellipsoid definition (Bolsterlee et al. 2014; Prinold and Bull 2014), four ellipsoids were defined, the centre of mass being the point of contact (i.e. the barycentre of the Angulus Acromialis, Angulus Inferior and Trigonum Spinae landmarks) (Figure 2). The axes of the ellipsoids were collinear to the thorax framework defined by the ISB (Wu et al. 2005).

(1) *6*-*DoF* – The scapula has free joints (six degrees-of-freedom, DoF). It corresponds to a segmental optimization approach.
(2) *NoEllips* – The scapula is included in an open-loop chain, i.e. without scapulothoracic constraint, with spherical joints at the acromioclavicular and glenohumeral joints.

**Table 1.** Description of the six kinematics models and their associated ellipsoid. (SC, AC, GH and ST joints mean sternoclavicular, acromioclavicular, glenohumeral and scapulothoracic joints and DoF stands for degrees of freedom. The ellipsoid parameters (in mm) are averaged over the subjects; *X*, *Y*, *Z* are the distances of the ellipsoid centre to the Incisura Jugularis landmark along the *X*, *Y*, *Z* axis; *Rx*, *Ry*, *Rz* are the radii along the *X*, *Y*, *Z* directions).

	SC joint	AC joint	GH joint	ST joint	Mean ellipsoid parameters (Avg±Std mm)
*6*-*DoF*	6-DoF	6-DoF	6-DoF	6-DoF	–
*NoEllips*	2-DoF	3-DoF	3-DoF	6-DoF	–
*BolsEllips*	2-DoF	3-DoF	3-DoF	3-DoF	X=-4.7±11.1;Y=-24.4±28.3;Z=105.6±27.5Rx=156±19.4;Ry=121.1±7.2;Rz=212.3±14
*PrinEllips*	2-DoF	3-DoF	3-DoF	3-DoF	X=69.8±9.2;Y=33.4±17.2;Z=96.9±24.3Rx=82.3±4.9;Ry=101.6±6.1;Rz=215.6±12.9
*ScapThoEllips*	2-DoF	3-DoF	3-DoF	3-DoF	X=20.1±11;Y=-4.1±22.3;Z=82.4±40.6Rx=153±18.9;Ry=139.5±24.2;Rz=363.7±80.8
*ScapLungEllips*	2-DoF	3-DoF	3-DoF	3-DoF	X=41.1±34.3;Y=29.7±31.8;Z=90.3±40.2Rx=125.4±37.1;Ry=166.4±43.4;Rz=391.8±67.6

The following four models derive from this second model with the inclusion of the scapulothoracic joint based on an ellipsoid.

(3) *BolsEllips* – A thorax-sized ellipsoid is added to the kinematic chain as defined in Bolsterlee et al. (2014). Briefly, the ellipsoid is scaled based on thoracic geometrical landmarks; it has to pass through the two most medial points of the thorax.
(4) *PrinEllips* – A lung-sized ellipsoid is defined as in Prinold and Bull (2014). Briefly, this ellipsoid is also obtained from geometrical observations: an ellipsoid corresponding to the Visible Human geometry is scaled using a height ratio (the subject’s height divided by Visible Human height). Then, a second scaling is performed so that the medial border of the scapula glides on the thorax without any penetrations for three arm attitudes.
(5) *ScapThoEllips* – Thorax-sized ellipsoid radii are obtained by minimizing the sum of the quadratic distances between the scapula landmarks (angulus inferior and trigonum spinae) and the ellipsoid envelope for the three arm attitudes described in Prinold and Bull (2014) (Equation 1). Furthermore, two equality constraints must be fulfilled: the most lateral point of the thorax must be the most lateral point of the mediolateral axis of the ellipsoid (Constraint *C*_1_ of Equation 2) and the centre of the ellipsoid must coincide with the origin of the centre of the ellipsoid must coincide with the origin of the thorax system of coordinates (Constraint *C*_2_ of Equation 2). The initial guess of this optimization was the *BolsEllips* ellipsoid.
(6) *ScapLungEllips* – A lung-sized ellipsoid is generated by optimization with the same cost function as the previous approach (Equation 1). The first equality constraint must also be fulfilled (Constraint *C*_1_ of Equation 2). The second constraint to be fulfilled is an inequality where the mediolateral component (*x*) of the most medial marker on the scapula must remain within a 20 mm distance of the centre of the ellipsoid (Constraint *C*_3_ of Equation 2). The initial guess of this optimization was the *PrinEllips* ellipsoid.

(1) $$\min_{\vec{E}}\left(\sum_{t=1}^{T=3}\sum_{i=1}^{N=2}D{\left(\vec{E},\vec{M_{\mathrm{ti}}\;}\right)}^{2}\right),$$

where *T* is the number of arm poses (*T* = 3), N is the number of geometrical markers (*N* = 2), *E* is a 6D vector including the ellipsoid parameters (the three radii and the XYZ-origin position), *M*_*ti*_ is the vector of the mean of the measured XYZ-position of the reflective marker *i* of the trial *t* and *D* is the Euclidian distance between each marker and the ellipsoid’s envelope (Cheshirekow 2009)*.*

(2) $$C_{1}\;:\left\{\begin{array}{c} C_{x\;}+R_{x}=\;{\text{ThL}}_{x}\\ C_{z}={\text{ThL}}_{z} \end{array}\right\}\;;C_{2}\;:\left\{C_{x}=0\right\}\;;\;C_{3}\;:\;\left\{\left|C_{x}-\frac{\text{ThL}}{2}\right|<20\right\},$$

where *C* and *R* are the centre and the radii of the ellipsoid and ThL is the most lateral point of the thorax.

#### Kinematics reconstruction and analysis

The joint kinematics of the 55 trials of each of the 15 subjects were reconstructed using a multibody inverse kinematic algorithm with the following cost function (Equation 3). For the two first kinematic chains (*6*-*DoF* and *NoEllips*), the second term of this cost function was omitted.

(3) $$\min_{q}\left(\sum_{i=1}^{N=35}M_{m_{i}}-M_{v_{i}}{\left(q\right)}^{2}+D{\left(\vec{E,\;}\vec{M_{\text{Ellips}}\left(q\right)\;}\right)}^{2}\right),$$

where **q** is the vector of the generalized coordinates driving the kinematic model, *N* is the total number of technical markers, *M*_*mi*_ and *M*_*vi*_ are the positions of the *i*^th^ measured and virtual markers, respectively, *M*_Ellips_ is the scapular contact point (barycentre of trigonum spinae, inferioris angulus and the centre point of the root spine) and *D* is the Euclidian distance between each marker and the ellipsoid’s envelope (Cheshirekow 2009).

To compare the data of the six reconstructed sets of kinematics to the scapula palpator results, the misorientation of the scapula was calculated (de Vries et al. 2010). To compute the scapula misorientation, the mean generalized coordinates of the reconstructed kinematics for each condition were converted to matrix of rotation (**R**_*S*_). Then the helical axis angle (*θ*) was calculated using the rotation matrix of the scapula palpator (**R**_*SL*_) of the corresponding trial (Equation 4) as follow:

(4) $$\theta ={\text{cos}}^{-1}\left(\frac{\text{trace}\left(R_{SL\;}^{T}R_{S}\right)}{2}\right),\;\theta \in \left[0,\;\pi \right]$$

As for statistical analysis, a repeated measures two-ways ANOVA was performed on the entire set of data, using IBM SPSS Statistics (2015), to evaluate the effect of the *kinematic chain* (6 models) and *arm elevation* (two groups of arm elevations were defined: *Low* elevations (below 90°) and *High* elevations (above 90°)) on scapula misorientation. If the ANOVA test was found to be significant, post-hoc tests with a Bonferroni correction were performed. The alpha level was set at 0.05.

## Results

On average (mean ± standard deviation), the minimum and the maximum angles of the scapula measured with the scapula palpator over the 55 poses of the 15 subjects were: 4.2 ± 11.1° and 55.5 ± 6.9° in internal-external rotation, −48.1 ± 9.8° and −1.2 ± 9.2° in upward–downward rotation and −7.5 ± 5.7° and 19.9° ± 11.9° in antero-posterior tilt.

**Figure 3.**
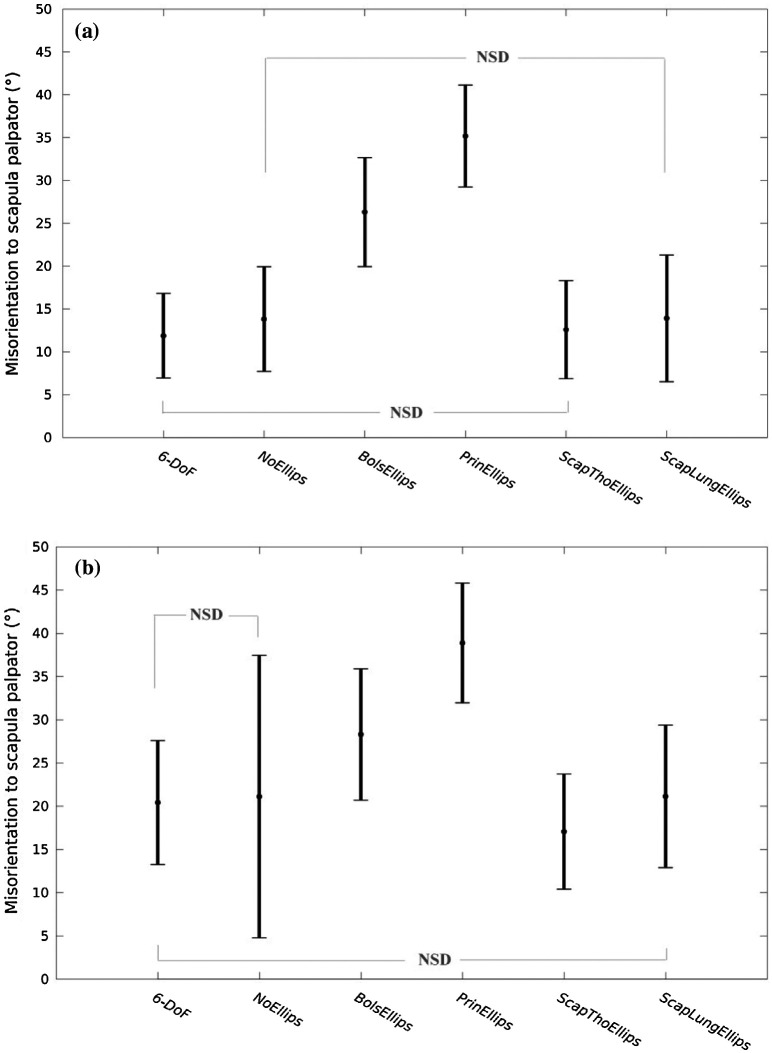
Mean and standard deviation of the scapula misorientation over all the trials of all subjects for the 6 approaches (*6*-*DoF*. *NoEllips*. *BolsEllips*. *PrinEllips*. *ScapThoEllips* and *ScapLungEllips*) at (a) low arm elevation (below 90°) and (b) high arm elevation (above 90°). The indication ‘NSD’ indicates if two approaches are not significantly different.

An interaction effect (model × elevation) was found by the two-ways ANOVA (*p* < 0.001). Post-hoc tests (Table 2) showed that *ScapThoEllips* was the most accurate approach both at low and high arm elevations. The *6*-*DoF* approach was not significantly different from *ScapThoEllips* at low elevations (below 90°; *p* > 0.05), while it was significantly different of 3.4° at high arm elevations. All the approaches were mostly significantly different from all the others (Figure 3) except for the 6-*DOF vs. ScapThoEllips* (as already mentioned) and the *NoEllips vs. ScapLungEllips* approaches at low arm elevations; and the 6-*DoF vs. NoEllips* as well as the 6-*DoF vs. ScapLungEllips* approaches at high elevations (*p* > 0.05). All the models showed larger scapula misorientations at high elevation than at low elevations (*p* < 0.001).

**Table 2.** Statistical results: estimated averages and standard scapula misorientations (°).

	Arm elevation	Average misorientation	Standard misorientation	95%-Confident interval	
				Lower limit	Upper limit
*6 DoF*	Low	11.9	0.3	11.3	12.4
	High	20.4	0.4	19.6	21.3
*NoEllips*	Low	13.8	0.3	13.1	14.5
	High	21.1	0.9	19.3	23.0
*BolsEllips*	Low	26.3	0.3	25.6	27.0
	High	28.3	0.5	27.4	29.2
*PrinEllips*	Low	35.2	0.3	34.5	35.9
	High	38.9	0.4	38.1	39.6
*ScapThoEllips*	Low	12.6	0.3	11.9	13.2
	High	17.1	0.4	16.3	17.8
*ScapLungEllips*	Low	13.9	0.4	13.1	14.7
	High	21.1	0.5	20.2	22.1

Regarding the scapula misorientation, the first approach, *6*-*DoF*, corresponding to a scapula segmental optimization, provided an averaged misorientation of 16.0 ± 7.1° (Figure 3). The *NoEllips* configuration, corresponding to an open-loop multibody optimization, generated a misorientation of 17.1 ± 12.0°. This standard deviation was the largest. Among the kinematic chains including a scapulothoracic joint, the ellipsoids generated by the *ScapThoEllips* and *ScapLungEllips* approaches were the most accurate since they generated the least amount of scapula misorientation compared to the scapula palpator kinematic measurements (14.9 ± 6.7° and 17.4 ± 8.3, respectively). The *BolsEllips* and *PrinEllips* approaches resulted in about twice as much misorientation: 28.1 ± 7.3° and 37.8 ± 6.7, respectively.

## Discussion

The objective was to determine which methods – including kinematic chain definition and ellipsoid scaling – are the most accurate to estimate scapular kinematics in a wide range of scapula and arm motions.

Two out of the six numerical methods provided results about as accurate (*6*-*DoF* and *ScapThoEllips*), even though the *6*-*DoF* results were slightly less accurate at high arm elevations than the *ScapThoEllips* results. These two methods vary greatly in terms of numerical complexity. The *6*-*DoF* approach is the easiest to implement since it corresponds to a basic segmental optimization introduced decades ago by Veldpaus et al. (1988) and refined by Cheze et al. (1995) and Challis (1995). On the other hand, *ScapThoEllips*, requires a preliminary optimization to generate the ellipsoid for the scapulothoracic joint and then a constrained non-linear least-squares algorithm to estimate the joint kinematics of a closed-loop chain. Since a segmental optimization (*6*-*DoF*) is much easier to implement, this method appears to be an interesting approach compared to multibody optimization for reconstructing scapula kinematics.

It had been shown in the literature (Bolsterlee et al. 2013; Seth et al. 2016), that both open-loop and closed-loop shoulder kinematic chains performed well for arm elevations below 90°. The present study agrees with and broadens this conclusion to movements implying large humeral and scapular range-of-motions. Indeed, the instruction given to the participants resulted in higher scapula range-of-motion, especially in internal-external rotation (averages went from 4 to 55°), than the ones observed during classic humeral elevation (averages went from 31 to 47° in the study by Ludewig et al. (2009)). In the present study, the open-loop multibody model (*NoEllips*) and closed-loop models (*ScapThoEllips*, *ScapLungEllips*) resulted in comparable averaged misorientation (about 16.5°, see Figure 3). Thus, an open-loop chain implemented in a multibody optimization is a possible method for scapula kinematic reconstruction at low arm elevations. However, the *NoEllips* approach showed a large standard deviation. This may result from the fact that the *NoEllips* algorithm failed to converge towards realistic configurations for three trials out of 825 (15 subjects × 55 poses) where upside-down scapula or scapula winged of 90° etc. were obtained. Thus, considering this lower robustness and the complexity of a multibody optimization compared to a segmental approach, the *NoEllips* approach is not as effective as the *6*-*DoF* approach.

Regarding the different approaches to build an ellipsoid to integrate a scapulothoracic joint, our results showed that making the ellipsoid fit the area browsed by the scapula (i.e. involving the scapula landmark positions in the optimization generating the ellipsoid) rather than the thorax geometry, improved the scapular kinematic reconstruction. The *ScapThoEllips* and *ScapLungEllips* approaches allowed to reduce almost by half the mean misorientation with respect to the scapula palpator compared to both geometrical ellipsoids (*BolsEllips* and *PrinEllips*). The gap between the scapula and the ellipsoid defined using the static pose may explain such discrepancies, since this gap is minimized during the genesis of the *ScapThoEllips* and *ScapLungEllips* ellipsoids only. The gaps of the *BolsEllips* (0.45 ± 0.08 mm) and *PrinEllips* (0.60 ± 0.10 mm) approaches were actually superior to the gaps of the *ScapThoEllips* (0.08 ± 0.04 mm) and *ScapLungEllips* (0.08 ± 0.06 mm)*.* However they remain very low, so the influence of this gap might be limited. When compared directly to their respective papers, *BolsEllips* and *PrinEllips* ellipsoids did not perform as well. For instance, Prinold and Bull (2014) reported reconstruction errors lower than 5° versus 30° in the current paper. Several reasons can explain this substantial difference. Firstly, Prinold and Bull (2014) studied pull-up task, while the current study investigates various poses where the scapula was at maximal amplitudes. Secondly, they reported their reconstruction errors as a set of three cardan angle errors, while here they are reported as a misorientation (unique angle associated to the helical axis), which gives, by definition, higher values. In fact, the misorientation angle cannot directly be compared to Cardan angles from previous studies. Since it takes into account the non-commutativity of matrix multiplication (Woltring 1991; Michaud et al. 2014), it is therefore a better estimation of the error between 3D attitudes. Furthermore, the kinematic chain used in this study (Jackson et al. 2012) was designed to reduce the kinematic reconstruction error of the whole upper limb and not specifically the scapular one. This may have introduced a reconstruction error at the scapula to the benefit of other segments. While this contribution to the reconstruction error would be hard to quantified, it should be similar for each model since all the approaches integrating a scapulothoracic joint used the same kinematic chain and thus it should not affect the overall conclusion. Finally, the scapula kinematics was reconstructed using skin markers, while scapula cluster was shown to be more accurate (van Andel et al. 2009). This choice was made since the use of an acromial cluster was limited to arm elevation under 100°. Once again, since all the approaches integrating a scapulothoracic were reconstructed using skin markers, this should not affect the current conclusions. In the literature, it was unclear which of the thorax- or lung-size ellipsoids were the best suited for scapular kinematic reconstructions. Our implementation of the thorax-size ellipsoids (*BolsEllips* and *ScapThoEllips)* gave better results that lung-size ellipsoids (*PrinEllips* and *ScapLungEllips*, respectively) at low and high arm elevations, scaling an ellipsoid of the thorax size should be preferred.

The intrinsic goal of segmental or multibody optimization based on skin markers is to accurately reconstruct the scapular kinematics during dynamic movements. Thus, a major limitation of this study is the absence of dynamic motions. Despite its great accuracy during static poses, the scapula palpator cannot be used in dynamic trials. Since soft tissue inertia can change the kinematics of the reconstructed bones, it can be risky to extrapolate the results reported here to dynamic movements. It is unlikely though that the overall finding of the study would drastically change. Further study using more invasive techniques to reconstruct bone kinematics will be needed to address this issue. A last limitation is that our focus was put only on the scapula misorientation. Assessing the accuracy of the other joints of the upper limb in function of the different kinematic models presented here could advantageously complete this work. Indeed, it would allow us to also know the influence of the kinematic chain on the joints of the entire upper limb.

## Conclusion

Regarding scapular kinematic reconstruction, the approach providing the most accurate results was a multibody optimization with a closed-loop chain (i.e. a chain integrating a scapulathoracic joint). The best model to represent the scapulathoracic joint was a point-to-ellipsoid contact with a thorax-size ellipsoid fitting the area browsed by the scapula. However, a segmental optimization of the scapula provided results similar to the results of this best-performing approach. Since segmental optimization is based on an algorithm much easier to implement, this method also appears to be appropriate for scapula kinematic reconstruction.

## Funding

This work was supported by the Natural Sciences and Engineering Research Council of Canada (NSERC) under [grant number RGPIN-2014-03912].

## Disclosure statement

No potential conflict of interest was reported by the authors.

## Supplemental data

Supplemental data for this article can be accessed at https://doi.org/10.1080/23335432.2017.1405741

## Supplementary Material

TBBE_1405741_Supplemental_Material.zip
