# Supplementary material for: Scapular kinematic reconstruction – segmental optimization, multibody optimization with open-loop or closed-loop chains: which one should be preferred?
Source: Int Biomech. 2017 Dec 15;4(2):86–94. doi: 10.1080/23335432.2017.1405741 (PMC7857462; doi:10.1080/23335432.2017.1405741)
Supplement: TBBE_1405741_Supplemental_Material.zip [file TBBE_A_1405741_SM8180.zip › Markers_SupplMat.docx]

**Supplementary Material: skin markers**


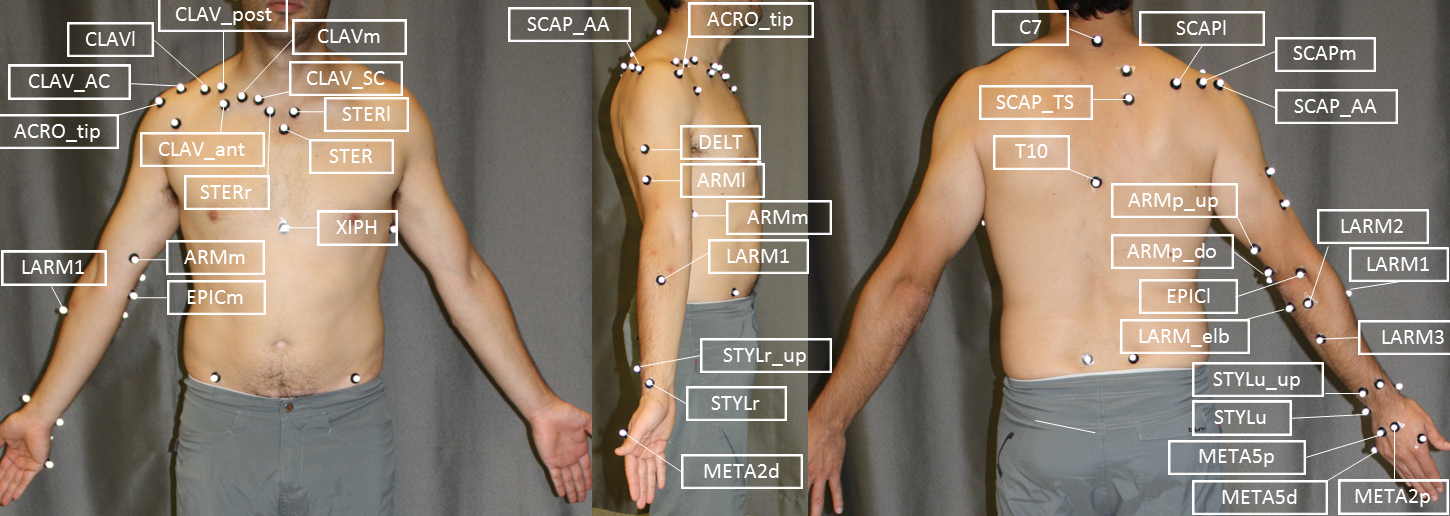


Supplementary Figure 1: Front, side and back views of a subject equipped with skin markers. The skin marker codes are detailed in the following table

Supplementary Table 1: List of the skin markers (* indicates that the marker isn’t illustrated on the previous figure)

| Area | Skin marker code | Bony landmark | Remarks |
| --- | --- | --- | --- |
| Thorax | STERr | Right clavicle, below CLAV_SC | Technical marker |
|  | STERl | Left clavicle, below CLAV_SC | Technical marker |
|  | STER | Sternum Notch | Technical & Anatomical marker |
|  | XIPH | Xiphoid Process | Technical & Anatomical marker |
|  | C7 | Cervical vertebrae 7 | Technical & Anatomical marker |
|  | T10 | Thoracic vertebrae 7 | Technical & Anatomical marker |
| Clavicle | CLAV_SC | Sternoclavicular joint | Anatomical marker |
|  | CLAVm | On the right clavicle | Technical marker |
|  | CLAV_ant | On the right clavicle | Technical marker |
|  | CLAV_post | On the right clavicle | Technical marker |
|  | CLAVl | On the right clavicle | Technical marker |
|  | CLAV_AC | Acromioclavicular joint | Anatomical marker |
| Scapula | ACRO_tip | Tip of the acromion | Technical marker |
|  | SCAP_AA | Acromion angle | Technical & Anatomical marker |
|  | SCAPl | On the scapula spine | Technical marker |
|  | SCAPm | On the scapula spine | Technical marker |
|  | SCAP_TS | Root of the scapula spine | Anatomical marker |
|  | SCAP_AI | Inferior angle of the scapula* | Anatomical marker |
| Arm | DELT | At the insertion of the deltoid | Technical marker |
|  | ARMl | Lateral to the biceps | Technical marker |
|  | ARMm | Medial to the biceps | Technical marker |
|  | ARMp_up | Above the olecranon | Technical marker |
|  | ARMp_do | Above the olecranon | Technical marker |
|  | EPICl | Lateral epicondyle | Technical & Anatomical marker |
|  | EPICm | Medial epicondyle | Technical & Anatomical marker |
| Forearm | LARM_elb | Underneath the olecranon, on the ulna | Technical & Anatomical marker |
|  | LARM1 | Anterior to the anconeus muscle | Technical & Anatomical marker |
|  | LARM2 | Posterior to the anconeus muscle | Technical & Anatomical marker |
|  | LARM3 | At the insertion of the anconeus muscle | Technical & Anatomical marker |
|  | STYLr | Radius styloid process | Technical & Anatomical marker |
|  | STYLr_up | 2 cm above STYLr | Technical & Anatomical marker |
|  | STYLu | Ulnar styloid process | Technical & Anatomical marker |
|  | STYLu_up | 2 cm above STYLu | Technical & Anatomical marker |
| Hand | META2d | Distal extremity of the 2^nd^ metacarpal bone | Technical & Anatomical marker |
|  | META2p | Proximal extremity of the 2^nd^ metacarpal bone | Technical & Anatomical marker |
|  | META5p | Proximal extremity of the 5^th^metacarpal bone | Technical & Anatomical marker |
|  | META5d | Distal extremity of the 5^th^metacarpal bone | Technical & Anatomical marker |
