# Supplementary material for: Scapular kinematic reconstruction – segmental optimization, multibody optimization with open-loop or closed-loop chains: which one should be preferred?
Source: Int Biomech. 2017 Dec 15;4(2):86–94. doi: 10.1080/23335432.2017.1405741 (PMC7857462; doi:10.1080/23335432.2017.1405741)
Supplement: TBBE_1405741_Supplemental_Material.zip [file TBBE_A_1405741_SM8180.zip › Results_SupplMat.docx]

**Supplementary Material: scapula orientations**

The following scapula rotations (Figures 1-3) were calculated from the transformation matrices between the scapula reference position (obtained with the scapular locator) and the scapula positions obtained with the 6 reconstruction methods.

| 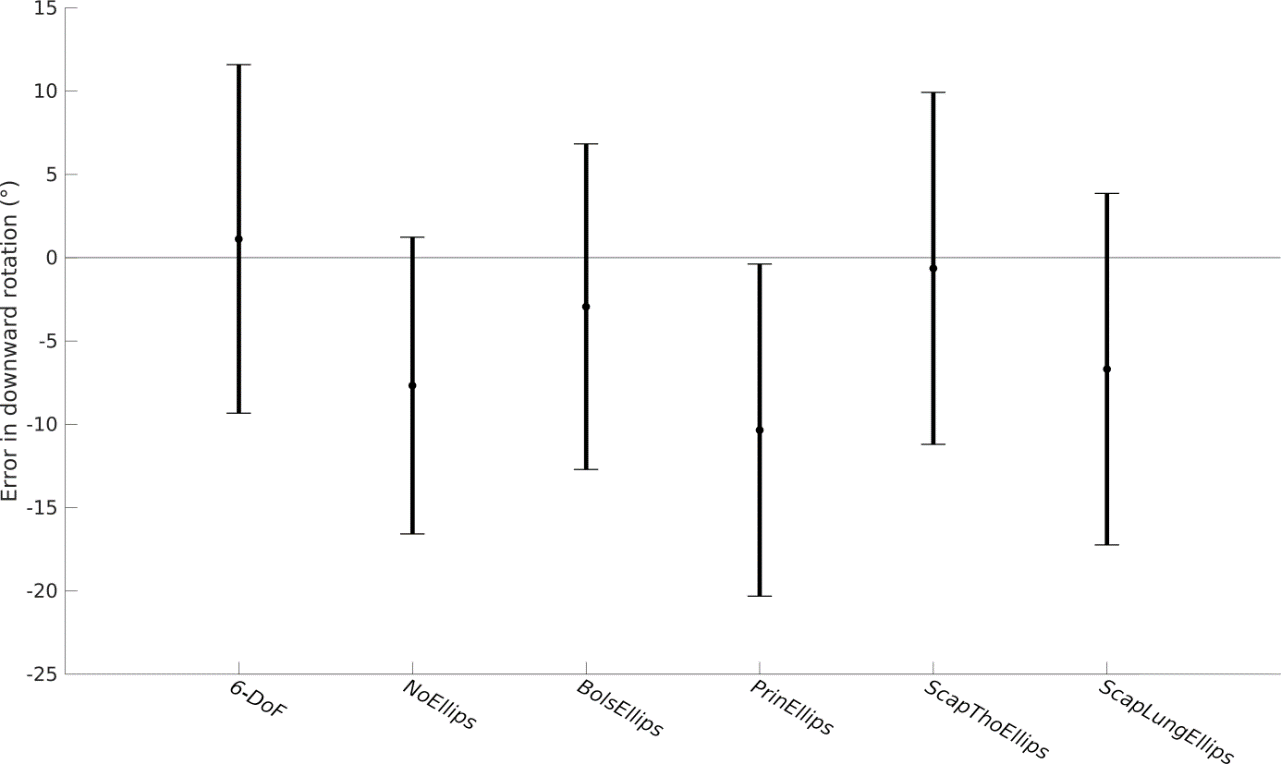 | 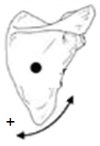 |
| --- | --- |

Supplementary Figure 2: Upward-downward rotation of the scapula. Average over all the postures of all the subjects ± standard deviation

| 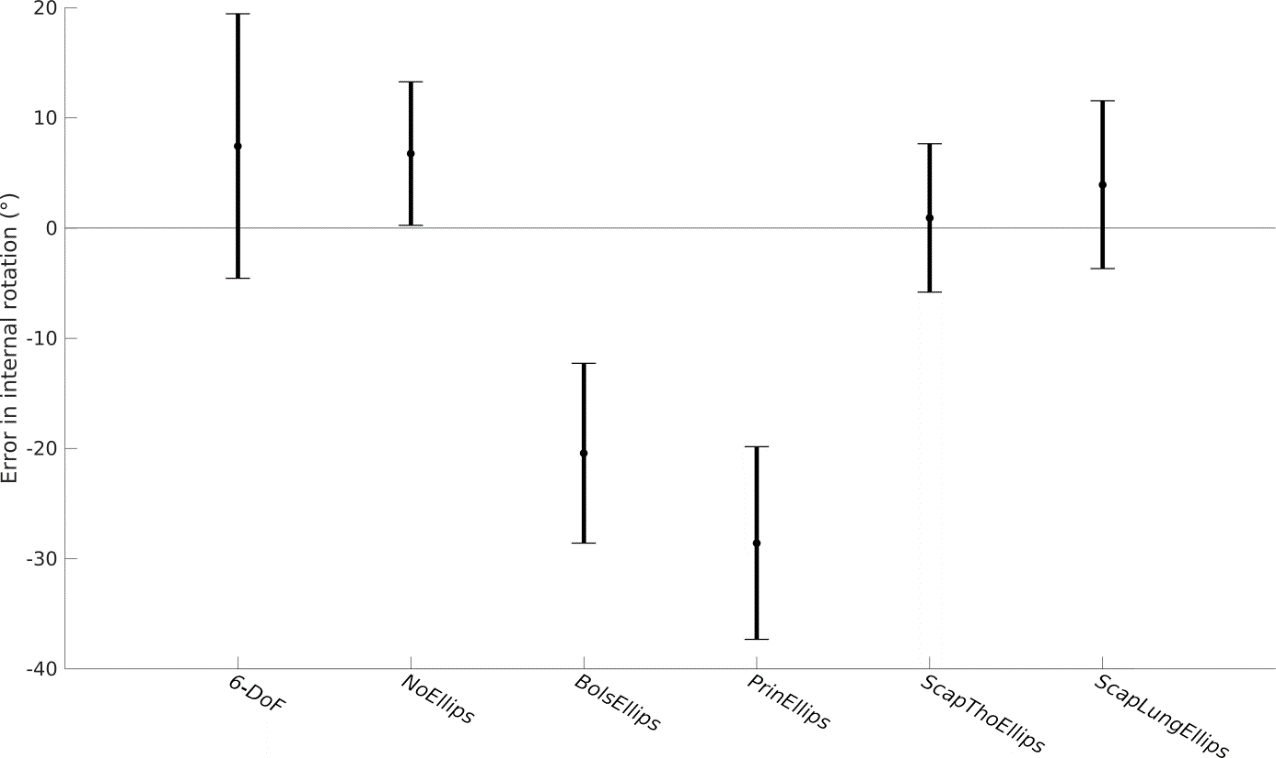 | 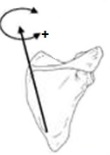 |
| --- | --- |

Supplementary Figure 3: Internal-external rotation of the scapula. Average over all the postures of all the subjects ± standard deviation

| 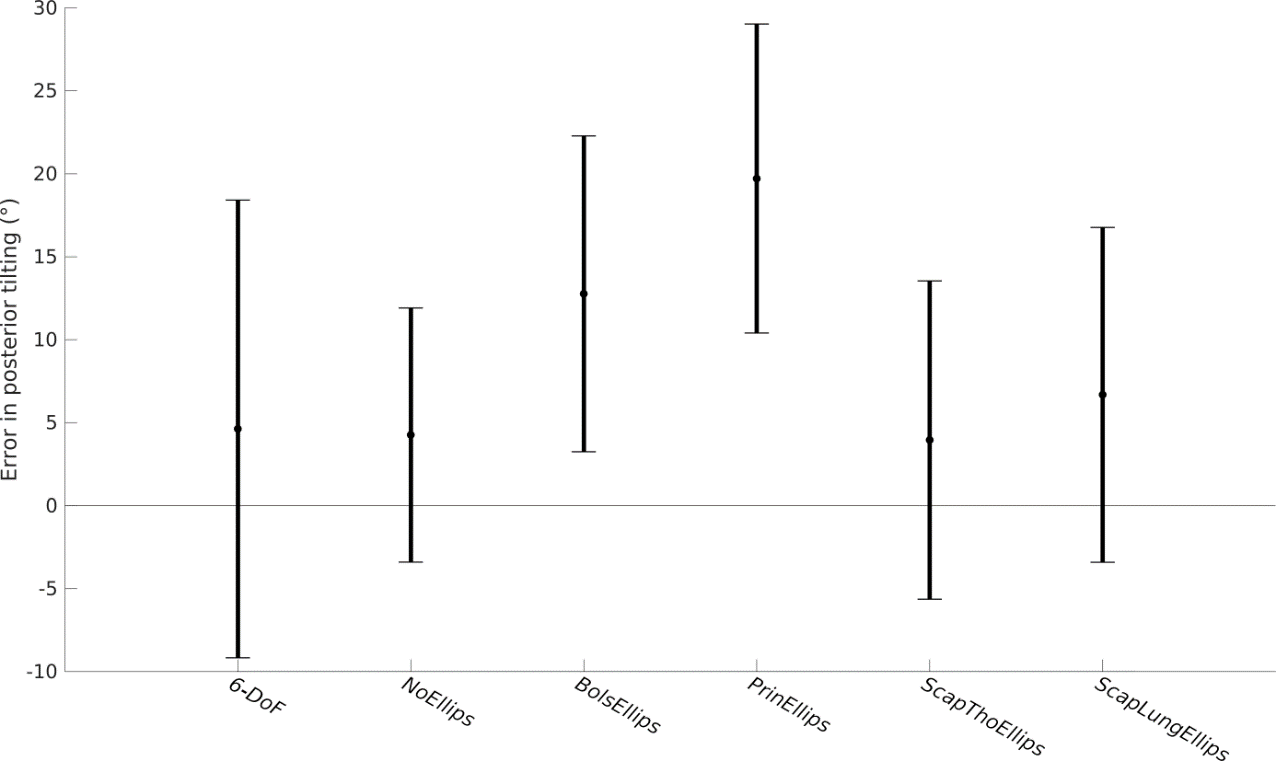 | 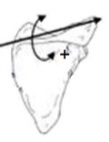 |
| --- | --- |

Supplementary Figure 4: Anterior-posterior tilt of the scapula. Average over all the postures of all the subjects ± standard deviation
